# Apples to apples comparison of standardized to unstandardized principal component analysis of methods that assign partial atomic charges in molecules

**DOI:** 10.1039/d2ra06349b

**Published:** 2022-11-03

**Authors:** Thomas A. Manz

**Affiliations:** Chemical & Materials Engineering, New Mexico State University Las Cruces New Mexico 88003-3805 USA tmanz@nmsu.edu

## Abstract

Articles by Cho *et al.* (*ChemPhysChem*, 2020, **21**, 688–696) and Manz (*RSC Adv.*, 2020, **10**, 44121–44148) performed unstandardized and standardized, respectively, principal component analysis (PCA) to study atomic charge assignment methods for molecular systems. Both articles used subsets of atomic charges computed by Cho *et al.*; however, the data subsets employed were not strictly identical. Herein, an element by element analysis of this dataset is first performed to compare the spread of charge values across individual chemical elements and charge assignment methods. This reveals an underlying problem with the reported Becke partial atomic charges in this dataset. Due to their unphysical values, these Becke charges were not included in the subsequent PCA. Standardized and unstandardized PCA are performed across two datasets: (i) 19 charge assignment methods having a complete basis set limit and (ii) all 25 charge assignment methods (excluding Becke) for which Cho *et al.* computed atomic charges. The dataset contained ∼2000 molecules having a total of 29 907 atoms in materials. The following five methods (listed here in alphabetical order) showed the greatest correlation to the first principal component in standardized and unstandardized PCA: DDEC6, Hirshfeld-I, ISA, MBIS, and MBSBickelhaupt (note: MBSBickelhaupt does not appear in the 19 methods dataset). For standardized PCA, the DDEC6 method ranked first followed closely by MBIS. For unstandardized PCA, Hirshfeld-I (19 methods) or MBSBickelhaupt (25 methods) ranked first followed by DDEC6 in second place (both 19 and 25 methods).

## Abstract 2

Standardized and unstandardized principal component analyses are directly compared for a dataset of ∼2000 molecules across various charge assignment methods.

## Graphical abstract

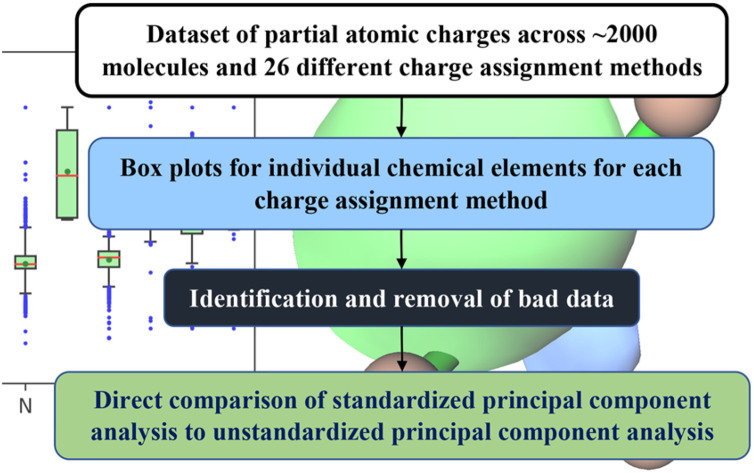

## 1. Introduction

Many factors should be considered when assessing the performance of methods for assigning partial atomic charges.^1–3^ Six factors that bear special consideration here include the following:

(1) The method should have a well-defined mathematical limit as the basis set is improved towards completeness (aka ‘a complete basis set limit’) and have atomic charge values that do not depend on the orientation of the external coordinate system (aka ‘rotational invariance’).^3^

(2) An assigned atomic charge should correspond to assigning some non-negative number of electrons to the atom. This means the assigned atomic charge should not exceed the atom's atomic number.

(3) Ideally, the method should work reliably across diverse material types including those containing both surface and buried atoms.

(4) The assigned atomic charges should exhibit similar values across similar chemical bonding environments (*i.e.*, good chemical and conformational transferability). While the precise definition of ‘similar chemical bonding environments’ may vary, one possible definition is based on the two chemical environments having the same bond connectivity graphs including first and second neighbors.^4^

(5) The assigned atomic charges should exhibit strong statistical correlations to related chemical and physical properties.

(6) The charge assignment method should be computationally efficient and convenient.

This article is primarily concerned with statistical correlations between different methods for assigning atomic charges. This relates to factor 5 above. Colloquially, one can think of analyzing correlations between different charge assignment methods as a form of democratic voting. The charge assignment method that exhibits the highest summed correlation to all charge assignment methods in the group has been ‘voted’ by the group to be the most representative of that group.

This ‘voting’ turns out to be far more important than one might naively expect. Rather than simply being a popularity contest, this ‘voting’ indicates which quantitative descriptor (*e.g.*, charge assignment method) is positioned to exhibit average or better statistical correlations to each of many related properties.^1^ An analogy is useful to understand how this works. Imagine a group of darts. The dart in the group's center always lands closer than approximately 50% or more of these darts to each and every conceivable target.^1^ Now if we have a group of methods for assigning atomic charges, a centrally located method would correlate better than approximately 50% or more of these methods to each of many properties related to atomic charges.^1^ This frees us from the bias of having to ‘choose’ which particular target property should be used to rank the charge assignment methods. This revolutionary idea is illuminated by the seven confluence principles that were recently introduced and proved.^1^

This turns out to be closely related to standardized principal component analysis (PCA), because the first principal component (*i.e.*, PC1) is defined as the normalized linear combination of standardized charge assignment methods that maximizes the sum of squared correlations between PC1 and all the charge assignment methods in the group.^1^ In standardized PCA, each independent descriptor (charge assignment method in this case) is standardized to have an average of zero and a variance of 1.^5^ This standardization gives each independent descriptor equal power to vote. In standardized PCA, the principal components are the eigenvectors of the correlation matrix. PC1 is the eigenvector with the largest eigenvalue, PC2 is the eigenvector with the second largest eigenvalue, and so on. The eigenvalues sum to the number of independent descriptors, N.

Unstandardized PCA gives a larger voting power to an independent descriptor having a larger variance. The average charge transfer magnitude of a charge assignment method equals its standard deviation, which is the square root of the variance.^1^ Hence, the QTAIM method (which has a large average charge transfer magnitude) receives more voting power than the Hirshfeld method (which has a small average charge transfer magnitude).^1^ In unstandardized PCA, the principal components are the eigenvectors of the covariance matrix. PC1 is the eigenvector with the largest eigenvalue, PC2 is the eigenvector with the second largest eigenvalue, and so on. The eigenvalues sum to the trace of the variance–covariance matrix (*i.e.*, the sum of variances of the independent descriptors). In unstandardized PCA, PC1 is defined as the normalized linear combination of charge assignment methods that maximizes its variance.

Cho *et al.* reported an unstandardized PCA on the covariance matrix of atomic charges computed by different atomic population analysis methods.^6^ There are two aspects of Cho *et al.*'s data analysis procedure that require reanalysis. As explained by Manz,^1^ a small number of bad datapoints were included in the unstandardized PCA of Cho *et al.* The nature of these bad datapoints was such that the reported atomic charges of a few charge assignment methods summed to the wrong system net charge for a handful of systems. Each of these bad datapoints was either corrected or not included in the standardized PCA of Manz.^1^

The second aspect that requires reanalysis is that Cho *et al.*'s presentation of the PCA results used different numbers of charge assignment methods on different pages of their journal article.^6^ Their complete dataset consisted of computed atomic charges for 26 different charge assignment methods applied to ∼2000 molecules from the GMTKN55 (ref. 7) collection. Table II on page 692 of their article shows the squared correlation matrix between 18 of these different charge assignment methods. Table III on page 693 lists the eigenvalues and first six principal component vectors for unstandardized PCA using 21 of these different charge assignment methods. Table IV on page 694 lists the squared correlation coefficient between individual charge assignment methods and PC1 for unstandardized PCA based on 16 of these different charge assignment methods.

Manz presented standardized PCA for the 20 of these different charge assignment methods that have a well-defined limit as the basis set is improved towards completeness.^1^ For comparison, he also presented standardized PCA that included all 26 charge assignment methods. Except for the correction/removal of a small number of bad datapoints as explained above and the somewhat differing numbers of charge assignment methods included in the PCA, Manz's standardized PCA used the same underlying dataset of molecules and computed atomic charges as Cho *et al.*

An apples to apples comparison between standardized PCA and unstandardized PCA results for this dataset is critically needed, because of the different conclusions reported by Cho *et al.* and Manz. For unstandardized PCA, Cho *et al.* reported on p. 688 of ref. 6: “The single charge distributions that have the greatest statistical similarity to the first principal component are iterated Hirshfeld (Hirshfeld-I) and a minimal-basis projected modification of Bickelhaupt charges.” For standardized PCA, Manz reported that the DDEC6 method had the highest correlation to the main principal component.^1^ As explained above, the datasets used in those two studies were not exactly equal. The main purpose of this article is to resolve this issue by providing a clean comparison between standardized and unstandardized PCA for the same dataset.

Another purpose of this article is to develop a better understanding of the large magnitude datapoints in this dataset. This will be done by examining the ranges and box plots for individual chemical elements and individual charge assignment methods. As discussed in the sections below, this produced some interesting and unexpected findings.

## 2. Methods

The parent dataset included the following 20 atomic charge assignment methods having a complete basis set limit:^1^ atomic charge partitioning (ACP),^8^ atomic dipole corrected Hirshfeld (ADCH),^9^ atomic polar tensor (APT),^3^ Becke,^10^ charges from electrostatic potentials using a grid (CHELPG),^11^ charge model 5 (CM5),^12^ sixth generation density-derived electrostatic and chemical (DDEC6),^13^ electronegativity equilibration charges (EEQ),^14^ Hirshfeld,^15^ intrinsic bond orbital (IBO),^16^ Hu–Lu–Yang electrostatic potential fitting (HLY),^17^ iterative atomic charge partitioning (i-ACP),^18^ iterative Hirshfeld (Hirshfeld-I),^19^ iterated stockholder atoms (ISA),^20^ minimal basis iterative stockholder (MBIS),^21^ minimal basis set Mulliken projection (MBSMulliken),^22^ Merz–Kollman electrostatic potential fitting (MK),^23^ quantum theory of atoms in molecules (QTAIM),^24^ restrained electrostatic potential fitting (RESP),^25^ and Voronoi deformation density (VDD).^26^ The parent dataset also included the following 6 charge assignment methods lacking a complete basis set limit:^1,6^ Bickelhaupt,^27^ minimal basis set Bickelhaupt projection (MBSBickelhaupt),^6^ Mulliken,^28^ natural population analysis (NPA),^29^ Ros-Schuit,^30^ and Stout-Politzer.^31^

Cho *et al.*'s quantum chemistry calculations used the PBE0 hybrid functional^32,33^ and the def2tzvpp^34^ basis set.^6^ They used geometries from the online GMTKN55 database^7^ without further optimization.^6^ Before bad datapoints were removed, Cho *et al.*'s dataset comprised 29 934 atoms-in-molecules for which atomic charges were reported; after Manz corrected/removed bad datapoints, 29 907 remained and were used in this work.^1^

In this work, PCA and data analysis were performed using Matlab. The Matlab ‘eig’ function was used to compute the eigenvalues and eigenvectors. Box plots were prepared using the Matplotlib utility in Python.

Quantum chemistry calculations of Li_4_C, SiF_4_, and AlF_3_ were performed using Gaussian 16 with geometry optimization.^35^ These geometries were converged such that the maximum force was less than 0.00045 hartrees bohr^−1^ and the maximum displacement was less than 0.0018 bohr. After geometry optimization, the DDEC6 atomic charges were computed for these molecules and found to match (within ±∼0.01 *e*) the values reported in Cho *et al.*'s dataset. The Foster-Boys^36^ localized orbitals of these molecules were prepared and plotted in Multiwfn^37^ (version 3.6).

Throughout this entire work, the unit for atomic charge is *e*, which is the absolute value of the charge of one electron.

## 3. Results and discussion

### 3.1 Elemental analysis of each charge assignment method to identify extreme atomic charges

Table 1 lists the number of atoms and charge range for each chemical element in the dataset. In Table 1, the number of atoms listed for each chemical element is per charge assignment method. The largest numbers of atoms were for H followed by C followed by O and N. The average, minima, and maxima values listed in Table 1 are for all of the data values across the listed chemical element and charge assignment methods. For example, across 26 charge assignment methods (including Becke), the 8917 × 26 = 231 842 carbon atom charge values had an average = −0.05, a minimum value = −6.73, and a maximum value = 7.46. These average, minimum, and maximum values are provided to give the reader a sense of the range of values present in the dataset.

**Table 1:** Elemental analysis of the dataset

Chemical element	Number of atoms	With Becke (26 charge methods)			Becke removed (25 charge methods)		
		Avg. atomic charge	Min. atomic charge	Max atomic charge	Avg. atomic charge	Min. atomic charge	Max atomic charge
Al	63	0.73	−1.57	2.59	0.75	−0.95	2.59
B	180	0.29	−8.78	2.49	0.30	−2.31	2.49
Be	14	0.41	−0.12	1.86	0.41	−0.12	1.86
Br	40	−0.06	−2.97	0.75	−0.05	−2.97	0.75
C	8917	−0.05	−6.73	7.46	−0.05	−4.02	2.56
Cl	241	−0.18	−2.62	2.68	−0.18	−2.62	2.68
F	414	−0.33	−1.60	1.67	−0.33	−1.60	1.67
H	15 616	0.15	−4.00	5.43	0.15	−1.00	1.51
Li	49	0.58	−0.79	1.06	0.59	−0.79	1.06
Mg	21	0.76	−0.10	1.93	0.77	−0.10	1.93
N	1478	−0.46	−3.17	8.24	−0.46	−2.28	1.97
Na	28	0.45	−2.00	1.04	0.45	−2.00	1.04
O	2294	−0.56	−3.09	1.66	−0.56	−1.84	1.00
P	220	0.38	−1.49	3.81	0.39	−1.49	3.81
S	225	0.05	−2.69	3.89	0.05	−2.69	3.89
Si	107	0.28	−1.29	3.33	0.29	−1.29	3.33
**All**	**29 907**	**0.0017**	**−8.78**	**8.24**	**0.0017**	**−4.02**	**3.89**

The charge ranges were unexpectedly large for Al, B, C, H, N, and O. If an atom loses all of its electrons, the largest physical charge it could have would equal its atomic number (*i.e.*, the number of protons in its nucleus). The maximum atomic charges of 5.43 for H, 7.46 for C, and 8.24 for N exceed this physical bound.

**Fig. 1:**
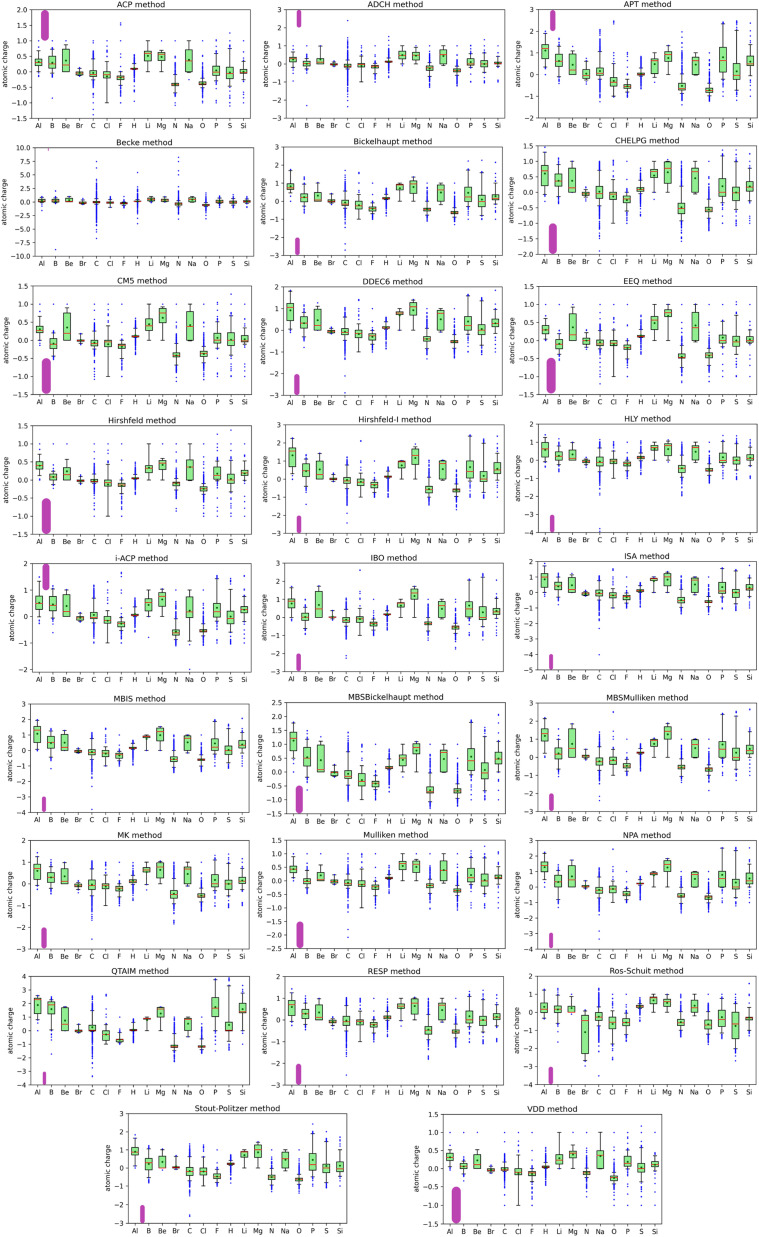
Boxplots for each charge assignment method showing for each chemical element the median atomic charge as a red line, the average as a darkgreen dot, the second and third quartiles in the lightgreen box, the 5th and 95th percentiles as whiskers, and blue dots for outside points. Each plot has a different *y*-axis scale. As a visual aid, the purple rounded rectangle has a length of 1.0 unit charge in each plot.

The last row in Table 1 refers to all of the chemical elements and represents the entire dataset. 29 907 was the total number of atomic charges reported per charge assignment method; the total number of numeric values in the dataset was 29 907 × 26 = 777 582. The listed overall average atomic charge value of 0.0017 is the average of these 777 582 data values; while this overall average atomic charge is informative, it does not represent anything other than the average of these 777 582 data values. While the average, minimum, and maximum values provide useful insights into the dataset, for fuller understanding of the dataset a more extensive statistical analysis is required and is provided by the box plots shown in Fig. 1. The data listed in Table 1 and the box plots shown in Fig. 1 are for the atomic charge values not standardized variables.

The box plots shown in Fig. 1 were prepared for each chemical element for each charge assignment method. The Becke charges showed an enormously large range with some unphysically large atomic charges for H, C, N that exceeded the atomic numbers of these chemical elements. Furthermore, some of the Becke negative atomic charges for B, H, and C had extremely large magnitudes that are not physically realistic. These results are not currently explainable. In the Becke method, electrons are assigned to each atom using Becke's multigrid integration weights^10^ for some chosen set of atomic radii. The Becke method was believed to be a stockholder-type^15^ electron density partitioning method that assigns atom-in-material electron densities *ρ*_A_[*r⃑*] using a non-negative atomic weighting function *w*_A_[*r⃑*]:

1 *w*_A_[*r⃑*] ≥ 0

2 *ρ*_A_[*r⃑*] = *w*_A_[*r⃑*]/*W*[*r⃑*]

3
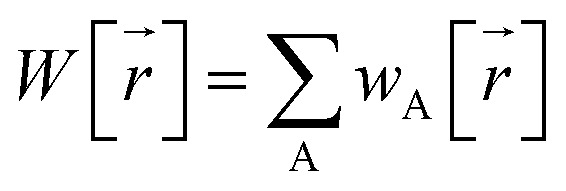


If this were true, then the Becke method should never assign a negative number of electrons to an atom in a material, and thus the Becke partial atomic charge should never exceed the atomic number. Since some of the Becke partial atomic charges for H, C, and N reported in Cho *et al.*'s dataset^6^ exceeded those elements' atomic numbers, there must be an underlying problem with how they were computed. Therefore, I had to remove all the Becke method data from this dataset when performing further statistical analysis.

The last three columns in Table 1 list the average, minimum, and maximum charge of each chemical element across the dataset of 25 charge assignment methods that does not include the Becke method. Except for H, the maximum atomic charge for each chemical element is now less than or equal to its atomic number. To better understand some of the atomic charges with large magnitudes, Table 2 lists details for each instance of a H atom having charge >1.00 and each instance of any other atom having a net charge larger in magnitude than 3.00. The ADCH and HLY methods gave some H atoms with charges >1.00; because these are not stockholder-type charge partitioning methods, they sometimes assign a negative number of electrons to an atom in a material. Several methods assigned atomic charges more negative than −3.00 to the C atom in CLi_4_ and/or CHLi_5_. The QTAIM method assigned atomic charges >3.00 to some of the P, S, and Si atoms in several molecules.

**Table 2:** Some atomic charges with large magnitudes: (a) H atom charges larger than +1.00 and (b) atomic charges having magnitudes larger than 3.00

Element	Atomic charges	Charge method	Systems
H	1.51, 1.30, 1.03	ADCH	AlB_2_C_2_FH_7_MgNO, C_18_H_22_N_4_O_14_P^a^
H	1.07	HLY	CHLi_5_
C	−3.93, −3.78	HLY	Li_4_C, CHLi_5_
C	−4.02	ISA	Li_4_C
C	−3.81	MBIS	Li_4_C
C	−3.35	NPA	Li_4_C
C	−3.38, −3.35	QTAIM	CHLi_5_, Li_4_C
C	−3.52	Ros-Schuit	CHLi_5_
P	3.14 to 3.81	QTAIM	41 different P atoms in various molecules
S	3.11 to 3.89	QTAIM	16 different S atoms in various molecules
Si	3.334, 3.327, 3.04	QTAIM	SiF_4_, AlBF_4_H_6_OSSi_2_

a One system with this stoichiometry had a ADCH charge of 1.30, while a different system with this same stoichiometry had a ADCH charge of 1.03.

Returning to the box plots in Fig. 1, the CM5, EEQ, Hirshfeld, and VDD methods gave the smallest ranges of atomic charges; atomic charges for these methods were between −1.5 and +1.5. The previously computed average charge transfer magnitudes for these molecular systems followed the order Hirshfeld < VDD < Mulliken < ACP < CM5 < ADCH < EEQ <⋯ < QTAIM.^1^ From these two observations, we conclude the Hirshfeld and VDD methods consistently give relatively small magnitudes of atomic charges. Behavior of the ISA method is interesting, because although its average charge transfer magnitude^1^ is moderate, sometimes it gives outliers with high magnitudes. For example, the atomic charge of C in Li_4_C was −4.02. If each Li atom only retained its core electrons the C atomic charge in this molecule would be −4. The ISA charge of −4.02 appears to indicate a slight loss of core electrons from the Li atoms, which seems physically dubious. For reasons that are not currently understood, for the Ros-Schuit method the Br atom box plot showed an extremely large range compared to the Br atom box plot for all of the other charge assignment methods.

For 16 of the charge assignment methods, the most negative C atom was in the Li_4_C molecule: ACP, Bickelhaupt, CHELPG, CM5, DDEC6, EEQ, Hirshfeld-I, HLY, IBO, ISA, MBIS, MBSMulliken, MK, NPA, RESP, and Stout-Politzer. For 19 of the charge assignment methods, the most positive Si atom was in the SiF_4_ molecule: APT, Bickelhaupt, CHELPG, DDEC6, Hirshfeld-I, HLY, i-ACP, IBO, ISA, MBIS, MBSBickelhaupt, MBSMulliken, MK, Mulliken, NPA, QTAIM, RESP, Ros-Schuit, and Stout-Politzer. For 14 of the charge assignment methods, the most positive Al atom was in the AlF_3_ molecule: Bickelhaupt, CHELPG, DDEC6, HLY, i-ACP, IBO, ISA, MBIS, MBSBickelhaupt, MBSMulliken, MK, NPA, RESP, and Ros-Schuit.

**Fig. 2:**
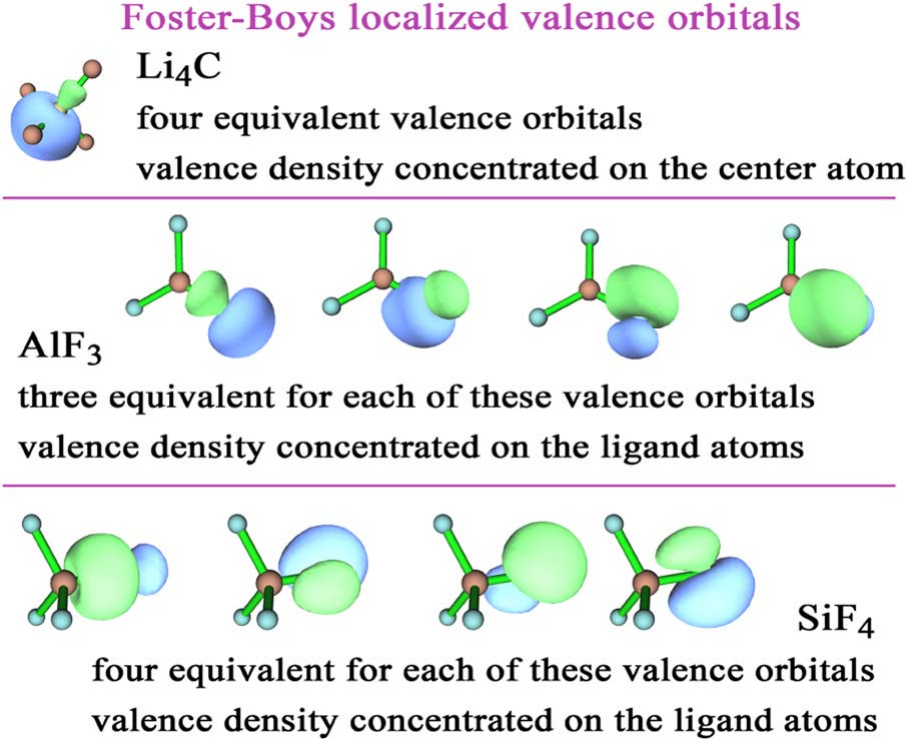
Foster-Boys localized orbitals for Li_4_C, AlF_3_, and SiF_4_ computed with the PBE0 exchange–correlation functional and def2tzvpp basis sets.

To better understand these results, Fig. 2 plots the Foster-Boys localized valence orbitals of Li_4_C, AlF_3_, and SiF_4_. In Li_4_C, these localized valence orbitals have a tetrahedral symmetry with most of the electron density located on the C atom; however, there is clearly some shared electron density in the bonding regions between the C and Li atoms. The AlF_3_ molecule is planar with most of the electron density of the valence orbitals located on the F atoms; however, there is clearly some shared electron density in the bonding regions between the Al and F atoms. The SiF_4_ molecule is tetrahedral with most of the electron density of the valence orbitals located on the F atoms; however, there is clearly some shared electron density in the bonding regions between the Si and F atoms. These results reflect the element electronegativity values. F is more electronegative than Al and Si.^38–40^ C is more electronegative than Li.^38–40^ These orbital plots show the DDEC6 computed atomic charges of −2.88 for C in Li_4_C, 1.84 for Al in AlF_3_, and 1.84 for Si in SF_4_ are plausible.

### 3.2 Comparing standardized to unstandardized PCA over identical datasets

In this section, standardized PCA is compared to unstandardized PCA over identical datasets. Although such a comparison is not revolutionary science, it nevertheless is a significant scientific advance in two respects. The prior studies of Cho *et al.* for unstandardized PCA and Manz for standardized PCA included some bad datapoints and were performed over somewhat different subsets of the same parent dataset.^1,6^ Cho *et al.*'s study included a small number of missing and bad datapoints that were corrected or removed in Manz's study.^1^ Moreover, both of those studies included the Becke data that are shown in the previous section to be erroneous. This raises the question of how robust the conclusions of those studies are to the removal of the bad datapoints.

As proved in ref. 1, for standardized PCA some performance measures are robust to corruption of any one of the independent descriptors. This robustness arises, because in standardized PCA each one of the independent descriptors contributes exactly 1.0 to the trace of the correlation matrix. For example, when performing standardized PCA over a set of 20 independent descriptors, each independent descriptor contributes exactly 5% to the trace of the correlation matrix: 1.0/20.0 = 5%. As a consequence, standardized PCA limits the potential impact that could be incurred by an error in one of the independent descriptors. This robustness is obviously a key advantage of using standardized PCA as opposed to using unstandardized PCA.

In unstandardized PCA, the contributions of different independent descriptors to the trace of the variance–covariance matrix can be different. Consequently, a large corruption of one of the independent descriptors can have an uncontrolled impact on the unstandardized PCA results. As shown in Section 3.1 above, the Becke charges in Cho *et al.*'s dataset were corrupted by a large amount. Because of this, it is not safe to assume that the unstandardized PCA results or conclusions that were reported by Cho *et al.*^6^ would automatically still hold once these bad datapoints are removed. Therefore, the conclusions of ref. 6 cannot automatically be assumed valid once it is discovered that some bad datapoints were included in their study.

In my view, the best way to resolve these issues is to reanalyze the dataset using both standardized PCA and unstandardized PCA with the bad datapoints removed. Such a reanalysis shows which of the previously proposed conclusions are valid and which are invalid (if any). It is absolutely essential to perform and publish such a reanalysis with the bad datapoints removed; otherwise, the unstandardized PCA results and conclusions that were reported by Cho *et al.*^6^ have to be set aside as inconclusive (*i.e.*, as no longer conclusive), because their validity cannot be established without such a reanalysis.

In addition to the issue of bad datapoints discussed above, a second issue that needs to be addressed is the previously published unstandardized PCA included a slightly different subset of charge assignment methods than the previously published standardized PCA. Cho *et al.* reported that the MBSBickelhaupt and Hirshfeld-I atomic charges were most strongly correlated to the PC1 of unstandardized PCA including 16 charge assignment methods; the DDEC6 and MBIS methods also exhibited almost as high of correlations to PC1.^6^ Manz reported that the DDEC6 atomic charges consistently exhibited the highest correlations to PC1 for standardized PCA across 20 methods with a complete basis set limit and across all 26 charge assignment methods; the MBIS, ISA, and Hirshfeld-I methods also exhibited almost as high of correlations to PC1. When including all 26 methods, the MBSBickelhaupt method also exhibited almost as high correlation to PC1 in standardized PCA as DDEC6 and MBIS.^1^ The second question that must be addressed is whether differences in the conclusions of those two studies is due to standardized *versus* unstandardized PCA or whether it is due to the use of slightly different datasets (*e.g.*, the inclusion of sligthly different subsets of charge assignment methods) for the analysis. The only way to definitively address this question is to perform unstandardized and standardized PCA on the same dataset and compare results.

Here, I performed standardized and unstandardized PCA on a dataset of 19 charge assignment methods having a complete basis set limit and across all 25 charge assignment methods. These datasets do not include the Becke method data. Table 3 summarizes the eigenvalues and the percentage of covariance (for unstandardized PCA) or correlation (for standardized PCA) accounted for by each principal component. In all cases, PC1 accounted for between 84.5% to 87.8% of the covariance or correlation while PC2 accounted for ≤7.1%. When using 19 charge methods with a complete basis set limit, PC2 in standardized PCA accounted for less than one variable's worth of correlation. On the other hand, when included all 25 charge methods, PC2 in standardized PCA accounted for 1.06 variable's worth of correlation; thus, PC2 may be considered significant in this case.

**Table 3:** PCA eigenvalues and percent of covariance or correlation explained (in parentheses) by each principal component

	Charge methods	PC1	PC2	PC3	PC4	% applies to
Unstandardized	19	1.835 (86.7%)	0.151 (7.1%)	0.060 (2.8%)	0.020 (1.0%)	Covariance
Standardized	19	16.683 (87.8%)	0.811 (4.3%)	0.554 (2.9%)	0.307 (1.6%)	Correlation
Unstandardized	25	2.502 (84.5%)	0.201 (6.8%)	0.096 (3.2%)	0.070 (2.4%)	Covariance
Standardized	25	21.678 (86.7%)	1.059 (4.2%)	0.616 (2.5%)	0.445 (1.8%)	Correlation

Coefficients for the first three principal components and correlation of each charge method to PC1 are listed in Table 4 (19 methods unstandardized PCA), Table 5 (19 methods standardized PCA), Table 6 (25 methods unstandardized PCA), and Table 7 (25 methods standardized PCA). Using the same notation as in ref. 1, the *k*th principal component's value for the *i*th datapoint (*i.e.*, *P*^(*k*)^_*i*_) is the following normalized linear combination of the various independent descriptors:

4 *P*^(*k*)^_*i*_ = *C*^(*k*,*j*)^*X*^(*j*)^_*i*_

5
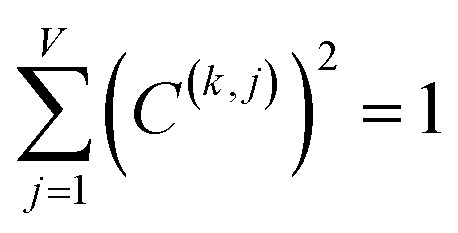
where *C*^(*k*,*j*)^ is the coefficient for independent descriptor *j* in the *k*th principal component, and *X*^(*j*)^_*i*_ is the value of independent descriptor *j* for the *i*th datapoint. In this work, there are 29 907 datapoints representing the different atoms in materials. In this work, the independent descriptors are the different methods for assigning atomic charges (*e.g.*, DDEC6, Hirshfeld, QTAIM, VDD, *etc.*). The total number of independent descriptors (*e.g.*, the number of different charge assignment methods) included in the PCA is V, and eqn (5) is the corresponding normalization condition for the *k*th principal component.^1^ If {*X*^(*j*)^} are unstandardized variables, the corresponding PCA is called unstandardized PCA. If {*X*^(*j*)^} are standardized variables, the corresponding PCA is called standardized PCA. As evident from the results presented in Tables 4–7, the values of the coefficients {*C*^(*k*,*j*)^} are generally different for standardized PCA compared to unstandardized PCA.

**Table 4:** Principal component coefficients for unstandardized PCA of 19 charge assignment methods having a complete basis set limit. In the first four columns, the methods are ordered from largest to smallest coefficient in PC1. In the last two columns, the methods are ordered from largest to smallest correlation to PC1. The last column lists the correlation of each charge assignment method to PC1

Charge method	PC1 coefficient	PC2 coefficient	PC3 coefficient	Charge method	Correlation to PC1
QTAIM	0.414	0.715	−0.151	Hirshfeld-I	0.983
MBSMulliken	0.294	−0.286	−0.483	DDEC6	0.982
MBIS	0.275	−0.128	−0.083	MBIS	0.980
Hirshfeld-I	0.274	−0.035	−0.133	ISA	0.980
APT	0.258	0.389	0.117	i-ACP	0.965
ISA	0.254	−0.057	0.185	CHELPG	0.953
HLY	0.234	−0.237	0.350	ACP	0.949
MK	0.230	−0.150	0.372	RESP	0.944
CHELPG	0.226	−0.044	0.362	MK	0.941
DDEC6	0.225	−0.104	−0.064	IBO	0.932
RESP	0.225	−0.139	0.355	MBSMulliken	0.919
IBO	0.222	−0.169	−0.314	HLY	0.916
i-ACP	0.213	0.109	0.050	CM5	0.912
ACP	0.155	−0.075	−0.065	EEQ	0.908
EEQ	0.154	−0.118	−0.128	Hirshfeld	0.901
CM5	0.150	−0.142	−0.120	VDD	0.899
ADCH	0.142	−0.209	−0.096	QTAIM	0.890
VDD	0.088	−0.007	−0.038	APT	0.886
Hirshfeld	0.085	−0.028	−0.051	ADCH	0.842

**Table 5:** Principal component coefficients for standardized PCA of 19 charge assignment methods having a complete basis set limit. The methods are ordered from largest to smallest coefficient in PC1. The last column lists the correlation of each charge assignment method to PC1. The rankings according to coefficient in PC1 and correlation to PC1 are identical

Charge method	PC1 coefficient	PC2 coefficient	PC3 coefficient	Correlation to PC1
DDEC6	0.242	0.028	0.000	0.987
MBIS	0.240	0.028	0.027	0.982
ISA	0.240	−0.090	0.172	0.978
Hirshfeld-I	0.239	−0.067	−0.044	0.975
ACP	0.236	0.087	−0.095	0.965
CHELPG	0.233	−0.126	0.331	0.953
i-ACP	0.233	−0.247	−0.059	0.953
RESP	0.233	−0.010	0.388	0.951
MK	0.232	−0.006	0.407	0.949
IBO	0.231	0.168	−0.182	0.943
CM5	0.231	0.253	−0.139	0.942
EEQ	0.228	0.211	−0.149	0.933
MBSMulliken	0.228	0.239	−0.155	0.932
HLY	0.227	0.098	0.437	0.929
Hirshfeld	0.226	0.051	−0.283	0.925
VDD	0.225	−0.022	−0.302	0.919
ADCH	0.216	0.381	−0.070	0.883
APT	0.208	−0.537	−0.123	0.848
QTAIM	0.206	−0.511	−0.221	0.842

**Table 6:** Principal component coefficients for unstandardized PCA of all 25 charge assignment methods. In the first four columns, the methods are ordered from largest to smallest coefficient in PC1. In the last two columns, the methods are ordered from largest to smallest correlation to PC1. The last column lists the correlation of each charge assignment method to PC1

Charge method	PC1 coefficient	PC2 coefficient	PC3 coefficient	Charge method	Correlation to PC1
QTAIM	0.344	−0.635	−0.396	MBSBickelhaupt	0.989
NPA	0.260	0.139	−0.005	DDEC6	0.985
MBSMulliken	0.259	0.219	0.006	MBIS	0.985
MBSBickelhaupt	0.239	−0.002	−0.068	Hirshfeld-I	0.982
MBIS	0.237	0.037	0.095	ISA	0.970
Hirshfeld-I	0.235	−0.045	0.072	Bickelhaupt	0.965
Stout-Politzer	0.230	0.204	−0.004	NPA	0.961
ISA	0.216	−0.048	0.157	ACP	0.959
APT	0.215	−0.370	−0.147	IBO	0.951
Bickelhaupt	0.204	0.051	−0.035	i-ACP	0.951
Ros-Schuit	0.200	0.500	−0.647	MBSMulliken	0.947
HLY	0.200	0.066	0.353	CHELPG	0.935
MK	0.194	0.001	0.295	RESP	0.932
DDEC6	0.194	0.022	0.098	CM5	0.931
IBO	0.194	0.108	0.033	MK	0.930
RESP	0.190	−0.002	0.277	EEQ	0.929
CHELPG	0.190	−0.075	0.218	Mulliken	0.929
i-ACP	0.180	−0.137	−0.038	Stout-Politzer	0.925
EEQ	0.135	0.098	−0.048	HLY	0.911
ACP	0.134	0.041	−0.001	Hirshfeld	0.904
CM5	0.131	0.105	−0.004	VDD	0.899
ADCH	0.125	0.146	0.067	QTAIM	0.864
Mulliken	0.117	0.089	0.054	ADCH	0.863
VDD	0.075	−0.008	−0.012	APT	0.859
Hirshfeld	0.073	0.006	0.008	Ros-Schuit	0.694

**Table 7:** Principal component coefficients for standardized PCA of all 25 charge assignment methods. The methods are ordered from largest to smallest coefficient in PC1. The last column lists the correlation of each charge assignment method to PC1. The rankings according to coefficient in PC1 and correlation to PC1 are identical

Charge method	PC1 coefficient	PC2 coefficient	PC3 coefficient	Correlation to PC1
DDEC6	0.212	−0.028	0.032	0.987
MBIS	0.211	−0.019	0.035	0.983
MBSBickelhaupt	0.211	−0.024	−0.126	0.982
Hirshfeld-I	0.209	−0.096	−0.053	0.974
ISA	0.208	−0.147	0.146	0.970
ACP	0.208	0.047	−0.031	0.967
Bickelhaupt	0.207	0.051	−0.134	0.966
NPA	0.206	0.123	−0.112	0.959
IBO	0.205	0.126	−0.089	0.955
MBSMulliken	0.204	0.201	−0.076	0.949
CM5	0.204	0.185	−0.005	0.949
Mulliken	0.203	0.150	0.031	0.947
i-ACP	0.202	−0.235	−0.114	0.942
EEQ	0.202	0.176	−0.052	0.942
RESP	0.202	−0.126	0.375	0.940
CHELPG	0.201	−0.211	0.288	0.938
MK	0.201	−0.124	0.390	0.938
Stout-Politzer	0.199	0.207	−0.078	0.928
Hirshfeld	0.198	0.003	−0.114	0.923
HLY	0.198	−0.045	0.447	0.922
VDD	0.196	−0.046	−0.158	0.915
ADCH	0.191	0.254	0.141	0.889
APT	0.179	−0.435	−0.284	0.835
QTAIM	0.178	−0.407	−0.375	0.830
Ros-Schuit	0.151	0.454	−0.186	0.701

For unstandardized PCA with 19 and 25 methods, the QTAIM method (which has the largest average charge transfer magnitude) had the highest coefficient in PC1 but relatively low correlation to PC1. The QTAIM method also had the largest magnitude coefficient in PC2. For unstandardized PCA with 19 methods having a complete basis set limit, the Hirshfeld-I, DDEC6, MBIS, and ISA methods had the highest correlation to PC1. For unstandardized PCA including all 25 methods, MBSBickelhaupt, DDEC6, MBIS, and Hirshfeld-I had the highest correlation to PC1. These results are roughly consistent with those reported by Cho *et al.* using a slightly different data subset derived from the same parent dataset, except that there is some minor reordering among the highly ranked methods.^6^

For standardized PCA, the rank of methods according to correlation to PC1 is always identical to the rank according to coefficient in PC1.^1^ For standardized PCA with 19 methods having a complete basis set limit, the DDEC6, MBIS, ISA, and Hirshfeld-I methods had the highest correlation to PC1. For standardized PCA including all 25 methods, the DDEC6, MBIS, MBSBickelhaupt, and Hirshfeld-I methods had the highest correlation to PC1. These rankings are identical to those when the Becke method is included, as previously reported in ref. 1. Specifically, rankings of the 19 methods in standardized PCA reported here are identical to those for the 20 methods reported in ref. 1, except the Becke method gets the last (*i.e.*, 20th ranking) when it is added to the dataset. With the exception of CM5 which is effectively tied with MBSMulliken, and i-ACP which is effectively tied with EEQ, the ranking of the 25 methods in standardized PCA reported here are identical to those for the 26 methods reported in ref. 1, except the Becke method gets the last (*i.e.*, 26th ranking) when it is added to the dataset.

For a more complete understanding of rankings in standardized PCA, Table 8 (19 methods) and Table 9 (25 methods) show the method rankings according to three additional ranking criteria: (a) the sum of correlations between all of the individual charge assignment methods and a particular charge assignment method,6
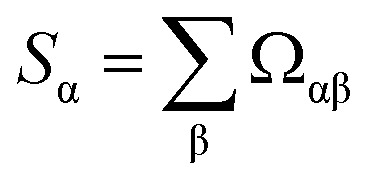


**Table 8:** Rank of each charge assignment method according to its amount of correlation to other charge assignment methods. The *S*_α_ and *Ω*[*α*,*ϕ*] ranking criteria always give the same order of methods. This table includes 19 charge assignment methods with a complete basis set limit

Rank	Method	*S*_α_	*Ω*[*α*,*ϕ*]	Method	Number (*Ω*_αβ_ > 0.8)	Method	Number (*Ω*_αβ_ > 0.9)
1	DDEC6	17.544	0.986	DDEC6	19	DDEC6	15
2	MBIS	17.455	0.981	MBIS	19	MBIS	14
3	ISA	17.401	0.978	ISA	19	Hirshfeld-I	11
4	Hirshfeld-I	17.345	0.975	Hirshfeld-I	19	ISA	10
5	ACP	17.159	0.965	i-ACP	18	ACP	9
6	i-ACP	16.960	0.953	CHELPG	18	i-ACP	9
7	CHELPG	16.946	0.953	ACP	17	CHELPG	9
8	RESP	16.909	0.951	RESP	17	RESP	8
9	MK	16.868	0.948	MK	17	MK	8
10	IBO	16.777	0.943	IBO	17	MBSMulliken	8
11	CM5	16.742	0.941	CM5	17	CM5	7
12	EEQ	16.585	0.932	EEQ	17	HLY	7
13	MBSMulliken	16.570	0.932	MBSMulliken	17	IBO	6
14	HLY	16.508	0.928	Hirshfeld	17	EEQ	6
15	Hirshfeld	16.458	0.925	VDD	17	Hirshfeld	3
16	VDD	16.367	0.920	HLY	16	APT	3
17	ADCH	15.692	0.882	ADCH	15	QTAIM	3
18	APT	15.124	0.850	APT	9	VDD	2
19	QTAIM	15.018	0.844	QTAIM	8	ADCH	1

**Table 9:** Rank of each charge assignment method according to its amount of correlation to other charge assignment methods. The *S*_α_ and *Ω*[*α*,*ϕ*] ranking criteria always give the same order of methods. This table includes all 25 charge assignment methods

Rank	Method	*S*_α_	*Ω*[*α*,*ϕ*]	Method	Number (*Ω*_αβ_ > 0.8)	Method	Number (*Ω*_αβ_ > 0.9)
1	DDEC6	22.915	0.986	DDEC6	24	DDEC6	20
2	MBIS	22.828	0.983	MBIS	24	MBIS	19
3	MBSBickelhaupt	22.811	0.982	MBSBickelhaupt	24	MBSBickelhaupt	16
4	Hirshfeld-I	22.615	0.973	Hirshfeld-I	24	Hirshfeld-I	14
5	ISA	22.532	0.970	ISA	24	ACP	14
6	ACP	22.474	0.967	Bickelhaupt	24	Bickelhaupt	14
7	Bickelhaupt	22.443	0.966	i-ACP	23	ISA	13
8	NPA	22.267	0.958	ACP	22	MBSMulliken	13
9	IBO	22.177	0.955	NPA	22	Mulliken	13
10	MBSMulliken	22.045	0.949	IBO	22	NPA	12
11	CM5	22.039	0.949	MBSMulliken	22	IBO	11
12	Mulliken	22.003	0.947	CM5	22	CM5	11
13	EEQ	21.887	0.942	Mulliken	22	i-ACP	10
14	i-ACP	21.887	0.942	EEQ	22	CHELPG	10
15	RESP	21.811	0.939	RESP	22	Stout-Politzer	10
16	CHELPG	21.775	0.937	CHELPG	22	EEQ	8
17	MK	21.763	0.937	MK	22	RESP	8
18	Stout-Politzer	21.550	0.928	Hirshfeld	22	MK	8
19	Hirshfeld	21.444	0.923	HLY	21	HLY	7
20	HLY	21.386	0.921	VDD	21	Hirshfeld	4
21	VDD	21.268	0.915	Stout-Politzer	20	APT	3
22	ADCH	20.657	0.889	ADCH	20	QTAIM	3
23	APT	19.411	0.836	APT	11	VDD	2
24	QTAIM	19.311	0.831	QTAIM	10	ADCH	1
25	Ros-Schuit	16.387	0.705	Ros-Schuit	1	Ros-Schuit	1

(b) the number of charge assignment methods having correlation *Ω*_αβ_ > 0.8 to a particular charge assignment method, and (c) the number of charge assignment methods having correlation *Ω*_αβ_ > 0.9 to a particular charge assignment method. As proved in ref. 1, ranking criterion (a) is equivalent to ranking the methods according to their correlation *Ω*[*α*,*ϕ*] to the average standardized variable *ϕ*. With some relatively minor differences, the rankings are approximately consistent between these three ranking criteria and the ranking according to correlation to PC1. The rankings in Table 8 (which does not include the Becke method) turned out to be identical to those reported in ref. 1 (which includes the Becke method), except the Becke method takes the last (*i.e.*, 20th place) when added. The rankings in Table 9 (which does not include the Becke method) turned out to be identical to those reported in ref. 1 (which includes the Becke method), except the Becke method takes the last (*i.e.*, 26th place) when added and there is a transposition of two adjacent methods (*i.e.*, CM5 and MBSMulliken) in the ranking according to *S*_α_.

Since there was a problem with the reported Becke atomic charges being incorrectly computed, no information is currently known about how the Becke atomic charges would perform if they would be computed correctly. The last ranking for the Becke method in ref. 1 may simply be a reflection of the fact that the reported Becke charges were computed incorrectly. Thus, this should not be taken as evidence that the Becke charge assignment method necessarily performs poorly if correctly implemented. To address the true performance of the Becke charge assignment method, an entirely new set of Becke charges would have to be computed across the molecular systems in this dataset. However, this is not feasible within the scope of present study, because Cho *et al.*'s dataset does not specifically give the *XYZ* coordinates of each atom along with the reported atomic charges. It is true the geometries were taken from the GMTKN55 collection, but matching the individual reported atomic charges to the individual geometries in the GMTKN55 collection would be tedious and not straightforward.

## 4. Conclusions

In prior literature, a detailed standardized PCA was performed on a slightly different dataset than a detailed unstandardized PCA, even though both datasets were derived from a common parent dataset.^1,6^ The slight differences in datasets made interpreting the differing conclusions of those two works difficult.

To address this issue, herein I compared standardized to unstandardized PCA for the same dataset of partial atomic charges computed across ∼2000 molecules using various charge assignment methods. This analysis was performed both for 19 charge assignment methods having a complete basis set limit and for all 25 charge assignment methods, which do not include the Becke method.

Analysis of maximum and minimum charge values together with box plots for each chemical element for each charge assignment method revealed important information. Most importantly, the reported Becke charges were found to be incorrectly computed. The Becke method is generally believed to be a stockholder-type charge partitioning approach that assigns a non-negative number of electrons to each atom in a material; however, the Becke charges reported by Cho *et al.*^6^ showed several instances of assigning negative numbers of electrons to atoms. Consequently, the Becke charge data was not included in the PCA of the present study.

Many of the charge assignment methods exhibited large charge magnitudes for the Li_4_C, SiF_4_, and AlF_3_ molecules. Each of these molecules has two chemical elements with a large electronegativity difference. To understand this behavior better, localized valence orbitals for these three molecules were plotted in Fig. 2. These localized valence orbitals showed high bond polarities with electron density concentrated on the more electronegative atom(s) and in the bonding regions between atoms.

The main takeaways from this work are as follows. First, standardized PCA yielded more consistent rankings both across different ranking criteria and with respect to adding or removing some methods from the analysis. Second, the following five methods (listed here in alphabetical order) showed the greatest correlation to the first principal component in standardized and unstandardized PCA: DDEC6, Hirshfeld-I, ISA, MBIS, and MBSBickelhaupt (note: MBSBickelhaupt does not appear in the 19 methods dataset). For standardized PCA, the DDEC6 method ranked first followed closely by MBIS. For unstandardized PCA, Hirshfeld-I (19 methods) or MBSBickelhaupt (25 methods) ranked first followed by DDEC6 in second place (both 19 and 25 methods).

For a proper context, the above conclusions of this work must also be considered in light of the following known properties (established in the prior literature not in this work) of these five charge assignment methods. MBSBickelhaupt is not recommended, because its atomic charges are sensitive to rotation of the external coordinate system.^1^ ISA often gives erratic results for materials with buried atoms.^1,41,42^ For molecules, the average charge transfer magnitudes follow the trend MBSBickelhaupt ≈ MBIS ≈ Hirshfeld-I > ISA > DDEC6 ≈ electrostatic potential fitting charges.^1^ DDEC6 charges have been more thoroughly tested and shown to work across a wider range of material types including many dense solids.^13,43^

## Conflicts of interest

There are no conflicts of interest to declare.

## Supplementary Material
